# Relationship between Pesticide Metabolites, Cytokine Patterns, and Asthma-Related Outcomes in Rural Women Workers

**DOI:** 10.3390/ijerph13100957

**Published:** 2016-09-27

**Authors:** Hussein H. Mwanga, Mohamed Aqiel Dalvie, Tanusha S. Singh, Kalavati Channa, Mohamed F. Jeebhay

**Affiliations:** 1 Division of Occupational Medicine and Centre for Environmental and Occupational Health Research, School of Public Health and Family Medicine, University of Cape Town, Room 4.47, Fourth Level, Falmouth Building, Anzio Road, Observatory, Cape Town 7925, South Africa; barakamwanga@gmail.com (H.H.M.); aqiel.dalvie@uct.ac.za (M.A.D.); 2 Department of Environmental and Occupational Health, School of Public Health and Social Sciences, Muhimbili University of Health and Allied Sciences, Dar es Salaam, Tanzania; 3 National Institute for Occupational Health, National Health Laboratory Services, Johannesburg 2000, South Africa; tanusha.singh@nioh.nhls.ac.za (T.S.S.); kalavati.channa@nioh.nhls.ac.za (K.C.); 4 Department of Clinical Microbiology & Infectious Diseases, School of Pathology, University of Witwatersrand, Johannesburg 2000, South Africa

**Keywords:** asthma, airway inflammation, cytokines, pesticide metabolites

## Abstract

The objective of this study was to investigate the relationship between exposure to organophosphate (OP) and pyrethroid (PYR) pesticides with serum cytokine patterns and asthma-related outcomes among rural women workers. A cross-sectional study was conducted among rural women (*n* = 211), including those working and living on farms and nearby town dwellers. Pesticide exposure was assessed using urinary metabolite concentrations of OP and PYR pesticides. Health outcome assessment was ascertained through the European Community Respiratory Health Survey (ECRHS) questionnaire, fractional exhaled nitric oxide (FeNO), and serum cytokines associated with asthma. The prevalence of doctor-diagnosed asthma was 11%, adult-onset asthma 9%, and current asthma 6%. In this population, the proportion of T helper type 2 (Th2) cytokines (interleukin (IL)-4, IL-5, and IL-13) detectable in subjects was between 18% and 40%, while the proportion of non-Th2 cytokines (IL-6, IL-8, IL-10, IL-17, and interferon gamma) was between 35% and 71%. Most Th2 and non-Th2 cytokines were positively associated with either OP or PYR metabolites. Non-Th2 cytokines showed much stronger associations with OP metabolites (Dimethyl phosphate OR = 4.23; 95% CI: 1.54–11.65) than Th2 cytokines (Dimethyl phosphate OR = 1.69; 95% CI: 0.83–3.46). This study suggests that exposure to most OP and some PYR pesticides may be associated with asthma-related cytokines, with non-Th2 cytokines demonstrating consistently stronger relationships.

## 1. Introduction

Several studies have reported an association between pesticides and various health effects, including adverse respiratory health outcomes such as asthma, rhinitis, and non-specific respiratory symptoms [1,2,3,4,5]. Various agents have been associated with these adverse respiratory health effects. These include organophosphate (OP) insecticides (diazinon, parathion, coumaphos, phorate, malathion, chlorpyrifos, terbufos, dichlorvos, and fonofos), carbamates (carbaryl), organochlorine insecticides (chlordane, heptachlor, lindane, and dichlorodiphenyltrichloroethane (DDT)), pyrethroid (PYR) insecticides (permethrin), herbicides [2,4,5-trichlorophenoxypropionic acid (2,4,5-TP), paraquat, diquat, ethyl dipropylthiocarbamate (EPTC), 2,4-dichlorophenoxyacetic acid (2,4-D), glyphosate, atrazine, chlorimuron-ethyl, imazethapyr, metolachlor, metribuzin, pendimethalin, alachlor, and trifluralin], fungicides (captan, metalaxyl, and benomyl) and fumigants (ethylene dibromide and 80/20 mix) [6,7,8,9,10].

The methods used to estimate exposures in previous studies have been generally crude and reliant on self-reported exposures, highlighting the need for more objective markers for individual pesticides [9]. Traditionally, acetylcholinesterase measurements in blood have been used as a proxy for estimating exposure to OP and carbamate insecticides [5]. With increasing laboratory analytical capabilities, several pesticide metabolites (commonly measured in urine samples) have also been used to estimate exposure to pesticides [11,12,13]. Despite the availability of these objective markers, only a few studies have investigated the relationship between pesticide metabolites in body fluids and asthma-related outcomes [13].

The pathophysiological mechanism of asthma associated with exposure to pesticides is heterogeneous and not clearly understood. However, there is increasing evidence for mechanisms suggesting an array of allergic inflammatory responses, irritant, as well as neuroendocrine responses [5,14,15,16].

This analysis is part of a larger study [5] of the respiratory health effects of pesticide exposure among rural women in the Western Cape province of South Africa. In this study, women with depressed cholinesterase (ChE) levels had increased odds of having allergic airway inflammation, based on the fractional exhaled nitric oxide (FeNO) determination. This finding suggested a probable association between exposure to ChE-depressing pesticides (OP and carbamate insecticides) and allergic asthma that needed further exploration.

The objective of this study was to determine urinary levels of OP and PYR pesticide metabolites and explore their association with various asthma-related outcomes, including cytokine pathways in order to identify possible pathophysiological mechanisms that may explain asthma-related endpoints.

## 2. Materials and Methods

### 2.1. Study Design, Population, and Sampling

A cross-sectional study of farm worker and resident women, as well as women residing in neighbouring towns in the Western Cape province of South Africa was conducted. About 100 women living on farms were targeted from the most accessible agricultural areas representative of the Western Cape farming population, and 100 women were targeted from neighboring towns that were about 5 to 10 km away from agricultural areas. 

Farm workers and residents were selected from the five to ten most accessible and representative farms in each area, and town women from the most accessible and representative houses in each area. One adult female participant per household was selected.

A total of 211 women were recruited into the study, including 113 women currently living on a farm (including 89 farm workers and 24 farm residents not working on the farm) and 98 residents in neighboring towns. Eight of the town residents actually worked on farms and were therefore classified as farm workers, which increased the number of farm workers in the study to 97 farm workers (89 women living on farms and 8 living in the nearby town). The additional 24 women residing but not working on farms were included in the category with the farm workers and were referred to as “farm dwellers” (*n* = 121), since based on a sub-analysis they had similar characteristics to that of the farm workers. The remaining 90 women who neither lived nor worked on a farm were referred to as “town dwellers”. The study was approved by the University of Cape Town’s (UCT) Research Ethics Committee (Reference 393/2009). Informed consent was obtained from participants prior to the interview. The study population and sampling methods have been previously described [5]. The study was conducted in accordance with the Declaration of Helsinki [17], and approved by the University of Cape Town’s Human Research Ethics Committee (Reference 393/2009). Written informed consent was obtained from participants prior to the study.

### 2.2. Questionnaire (Demographic Characteristics and Respiratory Symptoms)

The questionnaire included details on socio-demographic aspects, lifestyle factors and respiratory health, which was based on the abbreviated form of the standardised and validated European Community Respiratory Health Survey questionnaire [18]. The questionnaire was translated into Afrikaans and Xhosa and thereafter back-translated to ensure accuracy of the translation. Trained interviewers administered questionnaires to participants in the language of their choice.

Each participant’s height and weight were measured to calculate their body mass index (BMI).

### 2.3. Urinary Pesticide Metabolites

Spot urine samples (50 mL) were collected in plastic containers sealed with a plastic cap and kept on dry ice prior to being stored at −20 °C before being analysed. Urine samples were collected during the working week at the end of the work day for all subjects.

Dialkyl phosphate (DAP) metabolites (dimethylphosphate (DMP), dimethylthiophosphate (DMTP), dimethyldithiophosphate (DMDTP), diethylphosphate (DEP), diethylthiophosphate (DETP), and diethyldithiophosphate (DEDTP)) were measured according to the method by Hardt et al. [19], with slight modifications. 3,5,6-trichloropyridinol (TCPY) was measured according to the method described by Sams and Jones [20]. The PYR metabolites (*cis*- and *trans*-3-(2,2-dichlorovinyl)-2,2-dimethylcyclopropanecarboxylic acid (DCCA), *cis*-3-(2,2-dibromovinyl)-2,2-dimethylcyclopropanecarboxylic acid (DBCA), 4-fluorophenoxybenzoic acid (4F3PBA), and 3-phenoxybenzoic acid (3PBA)) were prepared according to the methodology of Arrebola et al. [21]. Pesticide metabolite levels were adjusted for urinary creatinine. Urine samples with creatinine concentrations within and outside the World Health Organization (WHO) recommended creatinine concentration range of 0.3 × 10^6^–3.0 × 10^6^ µg/L were distinguished and taken into account during analysis. Those levels outside the WHO range are not presented (*n* = 18).

### 2.4. Immunological Profile

#### 2.4.1. Phadiatop

All blood samples were collected at the end of the working day. A blood sample (9 mL) was drawn from each participant using a Becton Dickinson Vacutainer SST tube (with gel medium and clot activator) (BD Vacutainer Systems, Oxford, UK). The blood was allowed to clot for 1–2 h at room temperature (20–24 °C) and then centrifuged at 1350 rpm for 10 min at room temperature. The serum was initially stored at −20 °C before being transported on dry ice to be stored at −80 °C until assayed for further measurement.

The presence of sensitisation to common aeroallergens (house dust mite, grass pollen, cat, dog, and cockroach) was determined by the Phadiatop^®^ test (Thermo Fisher Scientific, Uppsala, Sweden). The Phadiatop^®^ test has a binary outcome, with a positive test indicative of the presence of sensitisation to any one of these common aeroallergens. In this study, atopy was defined as a positive Phadiatop^®^ test with a value of ≥0.35 kUA per litre.

#### 2.4.2. Serum Cytokines

Sera obtained from participants were also analysed for the presence of cytokines related to allergic and non-allergic asthma pathways. The BD^TM^ Cytometric Bead Array Human Inflammation kit (Becton Dickson, Oxford, UK) was used to quantitatively measure non-allergic inflammatory interleukin (IL-8, IL-6, IL-10, IL-17, interferon (IFN)-γ) protein levels in each sample. Six bead populations with distinct fluorescence intensities coated with the specific interleukins were multiplexed and resolved in the red channel of the flow cytometer BD FacsArray^TM^ (Becton Dickson, Oxford, UK). The inflammatory interleukins associated with allergic airway inflammation (IL-4, IL-5, IL-13) were measured using the BD^TM^ Cytometric Bead Array Human Soluble Protein Flex Set assay according to the manufacturer’s instructions (Becton Dickson, Oxford, UK). The assay allows for multiplexed analysis of multiple proteins from a single sample. The concentrations were determined using the FacsArray^TM^ (Becton Dickson, Oxford, UK). Standard curves of the standard serial dilutions, expressed by a four parameter logistic model (log CC = D + (A − D)/(1 + (log I/C)^B, where A = minimum asymptote, B = slope factor, C = inflection point, D = maximum asymptote), were used to determine the limit of detection (LOD) for each specific analyte (IL-4, IL-5, IL-6, IL-8, IL-10, IL-13, IL-17, IFN-γ) based on the average median fluorescent intensity (MFI) of the negative control. The concentration of analyte was expressed as picograms per millilitre (pg/mL). A regression coefficient (R^2^) for the standard curve for each analyte was accepted if ≥0.98. The assay detection limit for each analyte was determined by the average fitted concentration of the negative control (0 pg/mL) + 2 standard deviations for each analyte. The LOD for each cytokine was 0.10 pg/mL for IL-4, IL-5, IL-8, IL-13, and IFN-γ; 0.14 pg/mL for IL-6; 0.20 pg/mL for IL-10; and 0.22 pg/mL for IL-17.

### 2.5. Fractional Exhaled Nitric Oxide

FeNO measurement is a recognized non-invasive method for the assessment of allergic airway inflammation [22]. FeNO testing was conducted by a trained nurse. A hand-held portable nitric oxide sampling device (NIOX MINO^®^ Airway Inflammation Monitor (NIOX MINO); Aerocrine AB, Solna, Sweden) was used. Three technically adequate measurements were performed in accordance with the American Thoracic Society/European Respiratory Society recommendations [23]. FeNO test was carried out on all participants during the working week at the end of the working day. Special instructions were provided to workers to ensure that tested individuals did not smoke tobacco, eat, nor drink (at least 1 h before) prior to the test. The average value of three FeNO measurements conducted on each participant was used in the analysis.

### 2.6. Statistical Analysis

The exposure variables of interest comprised individual levels of OP metabolites, including dialkyl phosphates (DMP, DEP, DMTP, DMDTP, DETP and DEDTP), TCPY, and PYR metabolites (3PBA, 4F3PBA, DBCA, and *cis*- and *trans*-DCCA). Two additional variables were created that included the sum of all six DAP metabolites and the sum of all five pyrethroid metabolites. These metabolite levels were also dichotomised into a high- and low-exposure group, with the former having measurement values above the 75th percentile of the detected values.

The main defined outcome variables were doctor-diagnosed asthma, adult-onset asthma, current asthma, and high (>50 ppb) FeNO levels, detectable T helper type 2 (Th2), and non-Th2 cytokine profiles (all dichotomous variables). Adult-onset asthma was defined as a history of doctor-diagnosed asthma and having had the first asthma attack at the age of 16 years or later. Current asthma was defined as having had an attack of asthma in the last 12 months or currently taking medicines for asthma. Cytokine levels were dichotomised as detectable versus non-detectable to increase the statistical power [24].

The data was analysed using Stata statistical package version 12 [25]. Continuous variables were summarised using median and interquartile range, since not all variables were normally distributed. Wilcoxon sum rank test was used to assess the association between binary variables and continuous variables. Multivariate regression analyses (both logistic and linear) were used to examine the association between the outcomes of interest and pesticide metabolites, controlling for confounders such as current smoking, atopy, born on a farm, and level of education, as previously reported [5]. Confounders were selected on an a priori basis, according to biological plausibility, or using bivariate testing if *p* < 0.10. Atopy and current smoking were selected a priori for all outcomes. The covariates selected for inclusion in the final models in addition to these two variables were years of schooling and born on a farm, since both these variables had consistently strong associations (*p* < 0.05) with most outcomes in the bivariate analysis. Exposure variables were analysed separately for each model testing the different outcomes while adjusting for these covariates.

## 3. Results

### 3.1. Demographic Characteristics and Allergic and Asthma Outcomes

The demographic characteristics and the prevalence of asthma and allergic sensitisation outcomes are presented in Table 1. Overall, half of the study population were current smokers, with a higher proportion (57%) among farm dwellers. Town dwellers (median 12.33 ppb) had on average significantly higher FeNO levels compared to farm dwellers (median 9.17 ppb). Almost half (44%) of the study population were atopic. The prevalence of all asthma and allergic outcomes was higher among town dwellers, although this did not reach statistical significance.

### 3.2. Urinary Pesticide Metabolite Concentrations

Table 2 summarises the results of the urinary pesticide metabolites (adjusted for creatinine) detected in the study population. For some participants, urine samples were not sufficient to measure concentrations of the pesticide metabolites (7 samples for TCPY, 15 samples for dialkyl phosphates, and 10 samples for pyrethroid metabolites). Only 10 individuals (5%) had undetectable levels of pesticide metabolites (eight samples for TCPY, one sample each for DMTP and PYR metabolites). An alternative method of summarising data for the values below the LOD (by transforming the LOD value and dividing by the square root of two) did not result in any significant changes to the median and interquartile range from those estimated using the former method, which used only the detectable values. Urine samples from 18 participants (9%) were outside the WHO-recommended creatinine concentration range of 0.3 × 10^6^–3.0 × 10^6^ μg/L [26]. Most of the detected pesticide metabolites were higher among farm dwellers (range of difference: 0.03 to 6.72 µg/g of creatinine) except for the DMTP, DMDTP, and DETP, which were in contrast higher among town dwellers. TCPY and *trans*-DCCA were statistically significantly higher among farm dwellers. DMP and DMTP were the predominant OP metabolites detected, while 3PBA was the predominant PYR metabolite detected.

### 3.3. Serum Cytokine Concentrations

Table 3 presents detectable cytokine concentrations and the patterns observed in this population (*n* = 201). Blood samples could not be obtained from five subjects, and serum samples were insufficient for analysis from another five subjects. In general, Non-Th2 cytokines had higher median levels than Th2 cytokines. Th2 cytokine levels were, on average, higher among town dwellers, with IL-13 having a statistically significant higher level (*p* < 0.05). There was an inconsistent pattern for the non-Th2 cytokines, with some cytokines (IL-6 and IL-8) being higher among town dwellers while others (IL-10, IL-17, and IFN-γ) were higher among farm dwellers. The proportion of detectable Th2 cytokines were lower, and ranged from 18% to 40%. The proportion of detectable non-Th2 cytokines was almost two-fold higher, and ranged from 35% to 71%. Among the cytokines, IL-8 was the most dominantly detectable (71%) cytokine, and IL-5 the least detectable (18%). The proportion of detectable cytokines was not significantly different between town and farm dwellers.

### 3.4. Host-Related Attributes Associated with Asthma-Related Outcomes

Unadjusted logistic regression models were created to examine the relationship between individual host-related attributes and asthma-related outcomes. Age, BMI, current employment, and currently living on a farm were not associated with any outcome measure (Table 4). Higher level of education and being born on a farm were generally protective for the outcomes of interest. Atopy was positively associated with all asthma-related outcomes except the cytokines. Current smoking was predictably negatively associated with high FeNO (>50 ppb) (data not shown).

### 3.5. Association between Pesticide Metabolites and Asthma-Related Outcomes, Including Individual Cytokine Profiles in Multiple Regression Models

Table 5 and Table 6 summarise the results of adjusted multiple logistic regression models exploring the association between pesticide metabolites and different asthma-related outcomes. TCPY was weakly and positively associated with virtually all asthma, FeNO, and cytokine outcomes. Most organophosphate metabolites were associated with greater odds of having high FeNO levels (>50 ppb). However, there was no consistent association between pesticide metabolites and other outcomes. Both non-Th2 and Th2 cytokines were generally positively associated with pesticide metabolites, although non-Th2 cytokines (especially IL-8) showed stronger associations with Dimethyl phosphate (OR = 4.23; 95% CI: 1.54–11.65) and ethyl phosphate metabolites (DEP, DETP, and DEDTP). There was no consistent association between cytokine levels (continuous variables) and pesticide metabolites in multiple linear regression models (data not shown).

## 4. Discussion

This study indicated that OP and PYR metabolite levels in rural women in South Africa are higher than in the general population, confirming that exposure to these agents is related to extensive agrochemical use on farms [27,28,29,30,31,32]. Using these exposure proxy markers, the findings of this study suggest that a number of OP and some PYR pesticides are consistently positively associated with some asthma-related outcomes, which may be related to both Th2 and non-Th2 mechanisms, the latter pathway demonstrating consistently stronger relationships.

The DAP, TCPY, and PYR pesticide levels detected in this study were generally higher than levels found in the general population in other countries [27,28,29,30,31]. DAP levels were generally lower compared to levels among applicators and general farm workers in a previous study in South Africa [12], as well as pesticide applicators in Japan [33] and Italy [34]. A possible reason for the lower levels of DAP found in the current study compared to other studies [12,33,34] could be due to the very low number of pesticide applicators (only four) in the current study population, which was mainly comprised of general farm labourers. TCPY and PYR levels were generally of a similar or lower order of magnitude compared to farm workers and applicators in other settings [34,35]. To our knowledge, no previous study has measured TCPY—a marker of exposure to both chlorpyrifos and chlorpyrifos-methyl [34,36]—in farming or non-farming communities in South Africa. In our study, DBCA and 4F3PBA levels were higher compared to those detected in flight attendants [37], implying that our study participants were exposed to a greater extent to deltamethrin and cyfluthrin than the other pyrethroids.

In this study, individuals who lived in the surrounding towns near the farms were also exposed to OP and PYR pesticides, based on the results of the urinary determinations for pesticide metabolites. It is likely that these individuals who do not live on the farms may be exposed to pesticides either through pesticide drift suggested by our previous study [5], as a result of living in towns close to farming activities or other environmental exposures such as contaminated surfaces, food, and water, or through household use of pesticides [12].

Among the participants, IL-13 levels were significantly higher among town compared to farm dwellers. Moreover, town dwellers were more likely to be atopic, and a larger proportion (9%) had high FeNO (>50 ppb) levels compared to farm dwellers (6%). These findings suggest that atopic asthma was a more dominant entity in the town dweller group.

In this study population, a greater proportion of non-Th2 cytokines (71%) were detectable in the sera of participants than Th2 cytokines (52%). Furthermore, while OP and PYR metabolites were positively associated with both Th2 and non-Th2 cytokines, stronger associations were observed between non-Th2 cytokines and pesticide metabolites. This suggests that non-Th2 pathways may be playing a more dominant role in the airway inflammation observed in individuals exposed to these pesticides. Non-Th2 cytokines such as IL-8 and IL-17 have been linked to neutrophilic asthma [38], suggesting that this may be an important phenotype associated with OP and PYR pesticide exposures.

A more detailed analysis of cytokine patterns in the Th2 pathway also demonstrated that most OP and PYR metabolites were also positively associated with Th2 cytokines, suggesting an involvement of Th2 mechanisms in asthma-related outcomes. While the few case reports of asthma due to these pesticides previously reported in the literature [39,40,41,42] could not identify the pathophysiological mechanisms that were responsible, a more recently published study [15] reported the case of a 29 year old woman who developed anaphylaxis after exposure to pyrethroids, implying that these pesticides may well induce an allergic response. An association between pesticide exposure and allergic asthma has also been reported in other epidemiological studies [7,9].

The positive association observed between most of the OP metabolites and high FeNO in this study is consistent with the findings from our previous sub-study [5], which demonstrated a positive association between low levels of ChE and high FeNO (OR = 4.8; 95% CI: 0.80–28.00) as well as asthma symptom score (OR = 1.93; 95% CI: 1.09–3.44). However, the association between high FeNO and actual metabolites as demonstrated in this study was not as strong (OR = 2.53; 95% CI: 0.74–8.64). The latter finding could be due to the fact that the low ChE levels in the initial study may also have been related to carbamates in addition to OP exposures. Carbamates have also been reported to be associated with asthma [7], but were not measured in the current study due to resource constraints. The lack of a strong association between FeNO and pesticide metabolites could also imply that eosinophilic inflammation may not be a predominant mechanism in pesticide-related asthma and asthma-like disorders. This phenomenon has also been observed in studies of workers exposed to cleaning agents [24], suggesting other mechanisms (non-eosinophilic immunological and irritant mechanisms) could be playing a major role in asthma related to low molecular weight chemicals.

To our knowledge, this is the first study to investigate the effects of pesticide exposure (ascertained through urinary pesticide metabolite concentrations) on allergic and non-allergic airway inflammation (as determined by FeNO and cytokine pathways). There were, however, some limitations that need to be borne in mind in interpreting the results. The inability to demonstrate a strong association between the measured pesticide metabolites and some asthma-related outcomes could be attributable to the cross-sectional nature of this study, which may have introduced a healthy worker effect. Individuals who developed adverse respiratory health effects from pesticides may have left their employment on the farms to work in alternative jobs elsewhere. The short half-life of these OP and PYR metabolites—which are in fact markers of short-term exposure—may also have contributed to the inability to demonstrate an association, especially when related to the very specific and discrete “chronic” asthma-related variables such as doctor-diagnosed or adult-onset asthma. This is suggested from the findings that demonstrated much stronger associations with short-term sub-clinical outcomes such as serum cytokine levels than with the aforementioned variables. Furthermore, most of the biomarkers used in this study are not chemically specific, so they may represent exposure to a variety of pesticides, which may vary in their immunological toxicity. One cannot rule out the possibility that the associations observed between pesticide metabolites and cytokines could also be due to chance, since most pesticide metabolites were non-specific and may have arisen due to multiple tests of association being performed. Finally, due to the small sample size, the lack of power could have contributed to the dilution of the positive associations demonstrated.

Although not examined in this study, it is possible that irritant mechanisms played a major role in asthma among our study participants, since pesticides are known respiratory irritants [16]. This could also explain the lack of a strong association between pesticide metabolites and some asthma-related outcomes in this study, since irritant-induced asthma is usually a less severe form of the disease than immunological asthma [24,43].

## 5. Conclusions

In conclusion, this study has shown that most OP and some PYR pesticides are consistently positively associated with some asthma-related outcomes with distinguishable cytokine patterns. These cytokine patterns suggest that while non-Th2 cytokines play a greater role, the Th2 cytokines also seem to have a role in relation to pesticide exposures. Apart from these immunological (Th2 and non-Th2) mechanisms, it is important to emphasise that pesticides can also act as irritants and interact with functional irritant receptors, resulting in asthma [16]. However, the latter mechanisms were not explored in this study, and therefore merit further investigation. These results need to be explored in larger longitudinal studies with a greater representation of pesticide applicators and using more specific pesticide markers.

## Figures and Tables

**Table 1:** Demographic characteristics, allergic, and asthma outcomes among rural women (*n* = 211).

Characteristics	Farm Dwellers (*n* = 121)	Town Dwellers (*n* = 90)	Overall (*n* = 211)
**Demographic Characteristics: (Median, Interquartile Range)**			
Age (years)	33 (27–40) ***	40.5 (31–49)	37 (28-45)
BMI (kg/m^2^)	25.18 (21.57–30.81) **	28.58 (23.78–35.71)	26.44 (22.52–32.87)
Education (years of schooling)	9 (7–10)	9 (7–11)	9 (7–10)
Length of stay in current residence (years)	15 (8–24) **	22 (12–41)	17 (9–29)
Born on a farm: *n* (%)	83 (69) ***	13 (14)	96 (46)
Current smoker: *n* (%)	69 (57) *	36 (40)	105 (50)
Currently employed: *n* (%)	101 (84) ***	25 (28)	126 (60)
**Asthma-Related Outcomes: *n* (%)**			
Asthma attack in the last 12 months	2 (2)	1 (1)	3 (1)
Currently taking medicines for asthma	4 (3)	8 (9)	12 (6)
Current asthma	4 (3)	8 (9)	12 (6)
Doctor-diagnosed asthma	11 (9)	12 (13)	23 (11)
Adult-onset asthma	9 (7)	10 (11)	19 (9)
FeNO (ppb): median (interquartile range)	9.17 (5.67–14) **	12.33 (8.33–22.33)	10.33 (6–17.33)
FeNO > 50 ppb	7 (6)	8 (9)	15 (7)
**Allergic Sensitisation: *n* (%)**			
Atopy (positive Phadiatop)	46 (38)	44 (51)	90 (44)

* *p* < 0.05; ** *p* < 0.01; *** *p* < 0.001; BMI: body mass index; FeNO: fractional exhaled nitric oxide.

**Table 2:** Urinary pesticide metabolites levels among rural women.

Pesticide Metabolites	Farm Dwellers (*n* = 121)	Town Dwellers (*n* = 90)	Overall
Median (Interquartile Range)			
Corrected for Creatinine (µg/g Creatinine)			
**I. Organophosphate Metabolites (*n* = 178)**			
**Dialkyl Phosphates**			
∑DAP	141.42 (37.4–249.83)	132 (45.64–204.45)	133.59 (41.86–229.09)
DMP	32.91 (13.50–55.75)	26.19 (14.33–52.36)	29.63 (14.06–53.22)
DMTP (*n* = 177)	13.41 (3.05–62.45)	37.86 (6.55–77.20)	22.04 (4.53–65.85)
DMDTP	5.70 (0.83–51.51)	9.57 (0.87–66.22)	6.87 (0.85–61.77)
DEP	5.01 (1.37–12.90)	4.13 (0.59–9.47)	4.27 (1.08–10.04)
DETP	3.70 (1.15–26.98)	3.94 (1.35–26.18)	3.87 (1.20–26.98)
DEDTP	1.99 (0.55–5.10)	1.70 (0.60–8.02)	1.89 (0.58–6.44)
**TCPY**			
TCPY	6.35 (3.67–10.95) *	4.26 (2.72–8.27)	5.38 (3.25–9.45)
**II. Pyrethroid Metabolites (*n* = 182)**			
Pyrethroids	6.60 (3.61–9.96)	5.26 (2.74–8.42)	6.01 (3.24–9.67)
*Cis*-DCCA	0.72 (0.27–1.28)	0.56 (0.23–1.13)	0.63 (0.26–1.24)
*Trans*-DCCA	0.85 (0.48–1.29) **	0.59 (0.28–1.02)	0.70 (0.37–1.22)
DBCA	0.33 (0.05–0.63)	0.30 (0.04–0.60)	0.31 (0.05–0.62)
4F3PBA	0.76 (0.35–1.32)	0.70 (0.33–1.30)	0.73 (0.33–1.32)
3PBA	3.85 (2.13–6.25)	3.34 (2.27–5.92)	3.41 (2.21–6.00)

* *p* < 0.05; ** *p* < 0.01; TCPY: 3,5,6-trichloropyridinol; ∑DAP: sum of the six dialkyl phosphate metabolites; DMP: dimethyl phosphate; DMTP: dimethyl thiophosphate; DMDTP: dimethyl dithiophosphate; DEP: diethyl phosphate; DETP: diethyl thiophosphate; DEDTP: diethyl dithiophosphate; Pyrethroids: sum of the 5 pyrethroid metabolites; *cis*-DCCA: *cis*-2,2-dichlorovinyl-2,2-dimethylcyclopropane-1-carboxylic acid; *trans*-DCCA: *trans*-2,2-dichlorovinyl-2,2-dimethylcyclopropane-1-carboxylic acid; DBCA: *cis*-2,2-dibromovinyl-2,2-dimethylcyclopropane-1-carboxylic acid; 4F3PBA: 4-fluoro-3-phenoxybenzoic acid; 3PBA: 3-phenoxybenzoic acid.

**Table 3:** Serum cytokine levels among rural women (*n* = 201).

Cytokines	Distribution of Detected Values Median (Interquartile Range) in pg/mL			Limit of Detection (pg/mL)	Proportion Detected *n* (%)		
	Farm Dwellers	Town Dwellers	Overall		Farm Dwellers	Town Dwellers	Overall
**Th2 cytokines**							
**IL-4**	4.68 (3.20–6.92)	5.09 (3.52–8.12)	4.84 (3.52–7.25)	0.10	51 (43)	30 (37)	81 (40)
**IL-5**	2.06 (1.66–2.31)	2.23 (1.82–3.16)	2.07 (1.68–2.60)	0.10	23 (19)	13 (16)	36 (18)
**IL-13**	4.80 (2.98–6.37) *	6.06 (4.90–7.58)	5.55 (3.39–7.23)	0.10	39 (33)	28 (34)	67 (33)
**Any Th2 cytokine**	N/A			N/A	61 (51)	44 (54)	105 (52)
**Non-Th2 cytokines**							
**IL-6**	5.16 (3.61–7.50)	5.27 (3.75–10.26)	5.16 (3.62–8.69)	0.14	63 (53)	38 (46)	101 (50)
**IL-8**	16.49 (10.56–36.22)	19.47 (13.08–37.30)	18.93 (11.45–36.90)	0.10	85 (71)	57 (70)	142 (71)
**IL-10**	4.47 (2.80–5.60)	4.22 (2.74–6.36)	4.39 (2.77–5.87)	0.20	45 (38)	26 (32)	71 (35)
**IL-17**	10.34 (6.20–15.61)	9.13 (7.09–19.02)	9.76 (6.40–16.10)	0.22	52 (44)	28 (34)	80 (40)
**IFN-γ**	10.53 (7.41–17.80)	9.14 (6.56–17.30)	10.30 (7.41–17.30)	0.10	60 (50)	31 (38)	91 (45)
**Any non-Th2 cytokine**	N/A			N/A	85 (71)	57 (70)	142 (71)

IL: interleukin; IFN- γ: interferon gamma; * *p*-value < 0.05; Any Th2: any Th2 cytokines (IL-4, IL-5, or IL-13) detected; Any non-Th2: Any non-Th2 cytokines (IL-6, IL-8, IL-17, IL-10, or IFN-γ) detected; N/A: Not applicable.

**Table 4:** Unadjusted logistic regression models of the association between host-related attributes and asthma-related outcomes among rural women.

Host-Related Factors	Asthma-Related Outcomes: Odds Ratio (95% Confidence Interval)					
	Doctor Diagnosed Asthma	Adult Onset Asthma	Current Asthma	FeNO > 50 ppb	Any Th2 Cytokine Detected	Any Non-Th2 Cytokine Detected
**Prevalence (%) (*n* = 211)**	**11%**	**9%**	**6%**	**7%**	**52%**	**71%**
Age (years)	1.01 (0.98–1.05)	1.02 (0.99–1.06)	1.04 (0.99–1.09)	0.99 (0.95–1.04)	1.00 (0.97–1.02)	1.00 (0.97–1.02)
BMI (kg/m^2^)	1.02 (0.96–1.08)	1.03 (0.97–1.10)	1.01 (0.93–1.10)	1.02 (0.95–1.10)	1.01 (1.00–1.05)	1.02 (0.98–1.07)
Education (years of schooling)	0.85 (0.74–0.96)	0.85 (0.74–0.97)	0.78 (0.66–0.92)	1.18 (0.96–1.46)	0.98 (0.89–1.07)	1.02 (0.93–1.13)
Born on a farm	0.61 (0.25–1.50)	0.67 (0.25–1.79)	0.10 (0.01–0.79)	0.27 (0.07–0.99)	1.07 (0.62–1.87)	1.17 (0.63–2.15)
Current smoker	1.66 (0.68–4.02)	1.43 (0.55–3.72)	1.44 (0.44–4.70)	0.23 (0.06–0.84)	0.68 (0.39–1.19)	0.77 (0.42–1.41)
Currently employed	0.86 (0.36–2.07)	0.73 (0.28–1.87)	0.46 (0.14–1.50)	0.55 (0.19–1.58)	0.90 (0.51–1.59)	1.36 (0.74–2.53)
Atopy (positive Phadiatop)	3.37 (1.32–8.58)	3.10 (1.13–8.5)	7.12 (1.52–33.40)	21.28 (2.74–165.23)	1.09 (0.62–1.90)	1.32 (0.71–2.45)
Farm vs. town dwellers	0.65 (0.27–1.55)	0.64 (0.25–1.65)	0.35 (0.10–1.20)	0.61 (0.21–1.76)	0.91 (0.52–1.60)	1.10 (0.59–2.03)

Each odds ratio represents a separate unadjusted logistic regression model; Any Th2: any Th2 cytokines (IL-4, IL-5, or IL-13) detected; Any non-Th2: Any non-Th2 cytokines (IL-6, IL-8, IL-17, IL-10, or IFN-γ) detected. BMI: body mass index; FeNO: fractional exhaled nitric oxide.

**Table 5:** Adjusted multiple logistic regression models of the association between pesticide metabolites and asthma-related outcomes among rural women.

Pesticide Metabolites	Asthma-Related Outcomes: Odds Ratio (95% Confidence Interval)					
	Doctor-Diagnosed Asthma	Adult-Onset Asthma	Current Asthma	FeNO > 50 ppb	Any Th2 Cytokine Detected	Any Non-Th2 Cytokine Detected
**Prevalence (%) (*n* = 211)**	**11%**	**9%**	**6%**	**7%**	**52%**	**71%**
**I. Organophosphate Metabolites**						
**Dialkyl Phosphates**						
∑DAP	0.68 (0.23–2.05)	0.66 (0.20–2.20)	1.38 (0.33–5.76)	2.53 (0.74–8.64)	1.77 (0.90–3.46)	1.77 (0.81–3.87)
DMP	0.16 (0.02–1.29)	0.22 (0.03–1.72)	0.54 (0.06–4.83)	1.77 (0.40–7.88)	1.69 (0.83–3.46)	4.23 (1.54–11.65)
DMTP	0.12 (0.02–0.94)	0.16 (0.02–1.29)	ND	0.47 (0.10–2.15)	0.85 (0.42–1.72)	1.34 (0.60–3.00)
DMDTP	1.52 (0.53–4.33)	1.91 (0.62–5.82)	2.47 (0.60–10.13)	1.80 (0.45–7.23)	0.79 (0.39–1.62)	0.46 (0.22–0.98)
DEP	0.78 (0.23–2.62)	1.08 (0.31–3.71)	0.74 (0.13–4.31)	2.54 (0.62–10.37)	1.99 (0.95–4.19)	2.71 (1.05–7.00)
DETP	1.45 (0.46–4.60)	2.03 (0.61–6.73)	1.53 (0.26–8.97)	1.06 (0.23–4.87)	2.75 (1.27–5.92)	23.25 (3.08–175.49)
DEDTP	1.19 (0.38–3.78)	1.67 (0.51–5.45)	0.77 (0.13–4.51)	0.97 (0.22–4.33)	7.70 (3.00–19.74)	23.84 (3.15–180.74)
**TCPY**						
TCPY	1.35 (0.47–3.92)	1.41 (0.45–4.44)	1.26 (0.27–5.76)	1.50 (0.35–6.38)	1.56 (0.57–2.34)	1.93 (0.83–4.45)
**II. Pyrethroid Metabolites**						
Pyrethroids	0.62 (0.19–2.02)	0.84 (0.26–2.77)	2.04 (0.48–8.59)	0.35 (0.07–1.90)	1.32 (0.69–2.55)	2.18 (0.98–4.88)
*cis*-DCCA	0.11 (0.01–0.93)	0.16 (0.02–1.29)	0.23 (0.02–2.62)	0.59 (0.10–3.53)	1.47 (0.73–2.93)	2.10 (0.92–4.80)
*trans*-DCCA	0.14 (0.02–1.19)	ND	0.28 (0.02–3.47)	0.81 (0.12–5.31)	1.04 (0.52–2.10)	1.21 (0.56–2.63)
DBCA	0.17 (0.02–1.30)	0.22 (0.03–1.74)	ND	0.94 (0.20–4.36)	1.33 (0.66–2.67)	1.74 (0.78–3.88)
4F3PBA	1.01 (0.30–3.40)	1.40 (0.40–4.90)	1.74 (0.29–10.56)	1.07 (0.23–5.04)	2.51 (1.20–5.22)	4.32 (1.58–11.82)
3PBA	1.06 (0.35–3.19)	1.15 (0.35–3.76)	2.64 (0.61–11.47)	0.40 (0.07–2.31)	1.30 (0.64–2.64)	1.63 (0.72–3.69)

Adjusted for current smoking, atopy, born on a farm, and level for education. Each odds ratio represents a separate adjusted logistic regression model. Pesticide residues categorized as above 75th percentile of the detected values. ND: OR not calculable. Any Th2: any Th2 cytokines (IL-4, IL-5 or IL-13) detected; Any non-Th2: Any non-Th2 cytokines (IL-6, IL-8, IL-17, IL-10 or IFN-γ) detected. FeNO: fractional exhaled nitric oxide; TCPY: 3,5,6-trichloropyridinol; ∑DAP: sum of the six dialkyl phosphate metabolites; DMP: dimethyl phosphate; DMTP: dimethyl thiophosphate; DMDTP: dimethyl dithiophosphate; DEP: diethyl phosphate; DETP: diethyl thiophosphate; DEDTP: diethyl dithiophosphate; Pyrethroids: sum of the 5 pyrethroid metabolites; *cis*-DCCA: *cis*-2,2-dichlorovinyl-2,2-dimethylcyclopropane-1-carboxylic acid; *trans*-DCCA: *trans*-2,2-dichlorovinyl-2,2-dimethylcyclopropane-1-carboxylic acid; DBCA: *cis*-2,2-dibromovinyl-2,2-dimethylcyclopropane-1-carboxylic acid; 4F3PBA: 4-fluoro-3-phenoxybenzoic acid; 3PBA: 3-phenoxybenzoic acid.

**Table 6:** Adjusted multiple logistic regression models of the association between pesticide metabolites and individual inflammatory cytokines.

Pesticide Metabolites	Cytokines: Odds Ratio: (95% Confidence Interval)							
	Th2 Cytokines			Non-Th2 Cytokines				
	IL-4	IL-5	IL-13	IL-6	IL-8	IL-10	IL-17	IFN-γ
**I. Organophosphate Metabolites**								
**Dialkyl Phosphates**								
∑DAP	1.60 (0.83–3.11)	2.92 (1.33–6.43)	1.49 (0.76–2.93)	1.77 (0.91–3.44)	1.77 (0.81–3.87)	1.83 (0.93–3.60)	2.25 (1.16–4.37)	1.70 (0.88–3.27)
DMP	2.34 (1.14– 4.82)	2.00 (0.82–4.87)	1.38 (0.65–2.92)	3.69 (1.72–7.92)	4.23 (1.54–11.65)	2.65 (1.28–5.52)	3.58 (1.71–7.48)	3.25 (1.57–6.72)
DMTP	1.59 (0.77– 3.29)	0.84 (0.31–2.31)	0.49 (0.21–1.13)	1.76 (0.86–3.61)	1.34 (0.60–3.00)	1.06 (0.50–2.26)	1.53 (0.74–3.14)	0.85 (0.42–1.73)
DMDTP	0.39 (0.17–0.88)	0.28 (0.08–1.01)	1.39 (0.66–2.94)	0.21 (0.09–0.47)	0.46 (0.22–0.98)	0.27 (0.11–0.71)	0.25 (0.10–0.59)	0.39 (0.18–0.83)
DEP	3.50 (1.65–7.44)	7.76 (3.15–19.11)	1.87 (0.89–3.95)	3.43 (1.57–7.49)	2.71 (1.05–7.00)	7.79 (3.40–17.83)	4.59 (2.13–9.89)	3.63 (1.70–7.73)
DETP	4.09 (1.90–8.77)	8.15 (3.27–20.30)	2.35 (1.11–4.99)	5.85 (2.49–13.79)	23.25 (3.08–175.49)	4.18 (1.95–8.96)	8.07 (3.51–18.53)	4.92 (2.24–10.80)
DEDTP	10.28 (4.29–24.64)	13.26 (5.16–34.08)	4.69 (2.16–10.16)	14.87 (4.94–44.72)	23.84 (3.15–180.74)	19.13 (7.30–50.14)	17.05 (6.46–45.02)	25.12 (8.07–78.17)
**TCPY**								
TCPY	1.20 (0.58–2.48)	0.48 (0.15–1.47)	1.03 (0.48–2.22)	2.56 (1.23–5.34)	1.93 (0.83–4.45)	0.77 (0.35–1.68)	1.83 (0.89–3.74)	1.40 (0.69–2.83)
**II. Pyrethroid Metabolites**								
Pyrethroids	2.32 (1.19–4.52)	1.07 (0.46–2.50)	0.74 (0.36–1.50)	2.95 (1.48–5.90)	2.18 (0.98–4.88)	2.23 (1.14–4.37)	3.78 (1.91–7.50)	2.66 (1.36–5.19)
*cis*-DCCA	2.26 (1.12–4.58)	1.44 (0.59–3.49)	0.72 (0.33–1.57)	2.72 (1.32–5.58)	2.10 (0.92–4.80)	1.62 (0.78–3.34)	2.96 (1.45–6.03)	2.12 (1.06–4.24)
*trans*-DCCA	1.52 (0.75–3.11)	0.93 (0.36–2.43)	0.65 (0.29–1.47)	2.51 (1.21–5.20)	1.21 (0.56–2.63)	2.28 (1.10–4.73)	2.12 (1.04–4.33)	1.57 (0.78–3.17)
DBCA	1.60 (0.79–3.24)	1.03 (0.40–2.65)	0.76 (0.35–1.67)	2.40 (1.18–4.91)	1.74 (0.78–3.88)	1.80 (0.87–3.70)	2.09 (1.03–4.24)	1.63 (0.82–3.25)
4F3PBA	2.53 (1.23–5.19)	2.50 (1.04–6.00)	1.48 (0.70–3.13)	4.97 (2.24–11.04)	4.32 (1.58–11.82)	2.94 (1.41–6.13)	3.38 (1.63–7.02)	2.74 (1.34–5.61)
3PBA	2.68 (1.29–5.57)	0.95 (0.37–2.48)	0.83 (0.38–1.81)	2.18 (1.05–4.51)	1.63 (0.72–3.69)	2.50 (1.17–5.33)	3.79 (1.80–7.97)	2.17 (1.06–4.42)

Adjusted for current smoking, atopy, born on a farm, and level for education. Each odds ratio represents a separate adjusted logistic regression model. Pesticide residues categorized as above 75 percentile of the detected values. Cytokines categorised as detected vs. non-detected. ∑DAP: sum of the six dialkyl phosphate metabolites; DMP: dimethyl phosphate; DMTP: dimethyl thiophosphate; DMDTP: dimethyl dithiophosphate; DEP: diethyl phosphate; DETP: diethyl thiophosphate; DEDTP: diethyl dithiophosphate; Pyrethroids: sum of the 5 pyrethroid metabolites; *cis*-DCCA: *cis*-2,2-dichlorovinyl-2,2-dimethylcyclopropane-1-carboxylic acid; *trans*-DCCA: *trans*-2,2-dichlorovinyl-2,2-dimethylcyclopropane-1-carboxylic acid; DBCA: *cis*-2,2-dibromovinyl-2,2-dimethylcyclopropane-1-carboxylic acid; 4F3PBA: 4-fluoro-3-phenoxybenzoic acid; 3PBA: 3-phenoxybenzoic acid. IL: interleukin; IFN- γ: interferon gamma; TCPY: 3, 5, 6-trichloropyridinol.
